# Klippel-Feil Syndrome Associated with Renal and Cardiac Anomalies in an Infant: A Case Report

**DOI:** 10.31729/jnma.8303

**Published:** 2023-10-31

**Authors:** Dipa Yadav, Anish Bhattarai, Prakreeti Bhandari, Anu Danai, Umesh Kumar Singh

**Affiliations:** 1 Department of Paediatric Intensive Care, International Friendship Children's Hospital, Maharajgunj, Kathmandu, Nepal; 2 Department of Paediatrics, Nepalese Army Institute of Health Sciences, Sanobharyang, Kathmandu, Nepal

**Keywords:** *case reports*, *congenital*, *heart diseases*, *Klippel-Feil syndrome*, *scoliosis*

## Abstract

Klippel-Feil syndrome is a rare congenital bone disorder characterised by a triad of short neck, low posterior hairline and limited lateral bending of the neck with an annual incidence of 1 in 40,000 live births. It has remained an obscure term in the medical literature because of its variability in presentation and wide spectrum of anomalies involving multiple organ systems. It is unusual to find a case that has all three classical triad features. Here, we present a case of a 9-month-old infant who manifests not only all three classical triad features associated with Klippel-Feil syndrome but also demonstrates the presence of congenital heart disease, scoliosis, and renal ectopia. An early comprehensive evaluation of a suspected case is essential for diagnosis and counselling which impacts its prognosis, helps minimize social stigma and affords parents the opportunity to consider cosmetic surgery as an option, should they choose to pursue it.

## INTRODUCTION

Klippel-Feil syndrome (KFS) is a complex condition characterized by improper segmentation or congenital fusion of at least one vertebral motion segment of the cervical spine with or without additional spinal or extraspinal manifestations.^1^ First described in 1912, its classical triad includes a short neck, low hairline, and limited lateral bending of the neck.^2,3^ These features however collectively appear in less than half of all cases.^4^ Here, we report an infant with the complete classic triad of features. Remarkably, this infant also exhibits cardiac and renal anomalies, highlighting the syndrome's diverse impact on multiple organ systems.

## CASE REPORT

**Figure 1:**
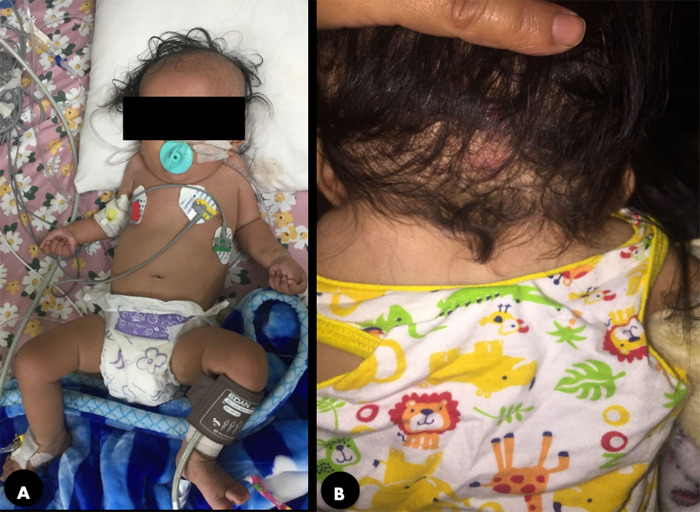
A) Short neck and, B) Low posterior hairline.

A 9-month-old female infant presented to the Emergency Department with a 15-day history of fever, fast breathing, and dry cough. She had a history of similar symptoms, requiring intensive treatment in the past at multiple different centres. She came to our hospital after 4 days of stay in the intensive care unit (ICU) of another rural hospital, with no improvement. Past medical history was significant for laboured breathing and excessive sweating after breastfeeding. Upon admission to our facility, a thorough physical examination showed tachycardia, elevated temperature and very high respiratory rate along with signs of distress, including subcostal recessions and nasal flaring. Chest auscultation revealed bronchial breath sounds on the right side. Cardiac examination revealed a Grade 3/6 systolic murmur with the highest intensity on the left second intercostal space along with splitting of the second heard sound. On head-to-toe examination, we observed a short neck and limited neck mobility, resulting in the appearance of a low hairline (Figure 1).

**Figure 2:**
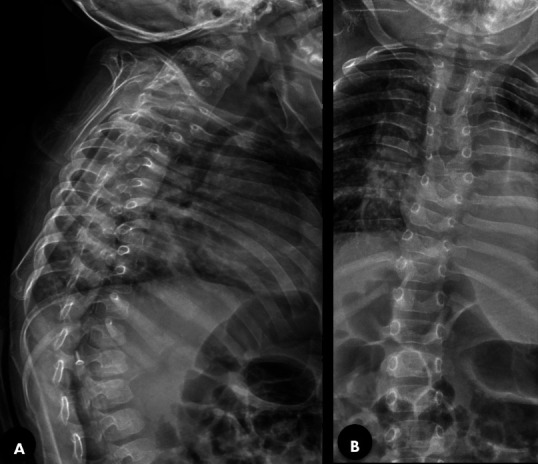
A) Cervicothoracic spine radiograph showing C3-C4 fusion, B) Thoracolumbar radiograph showing scoliosis.

In addition, an examination of her back revealed features of scoliosis. In the ICU, the infant received immediate high-flow oxygen therapy via bubble continuous positive airway pressure, appropriate antibiotics, and supportive care. Despite these interventions, her respiratory distress continued to deteriorate, requiring mechanical ventilation on the fourth day of hospitalization. The presence of a short neck with limited neck bending strongly suggested a rare spinal anomaly. Chest radiography performed during her ICU stay also showed a possible fusion of the cervical vertebrae. To confirm our suspicion, radiography of the cervical spine and thoracolumbar region was performed in anteroposterior and lateral views. The radiograph of the cervicothoracic (C-T) spine showed fusion of the C3-C4 vertebrae and the thoracolumbar radiograph showed scoliosis (Figure 2).

**Figure 3:**
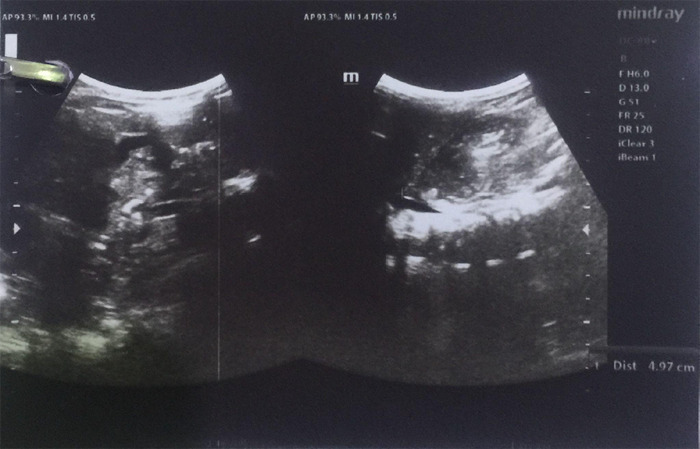
Cross-fused renal ectopia as seen on ultrasonography.

Hence, we were able to confirm that this was KlippelFeil syndrome. To look for any additional anomalies, we performed ultrasonography of the abdomen and pelvis, which showed crossed fused renal ectopia (Figure 3).

**Figure 4:**
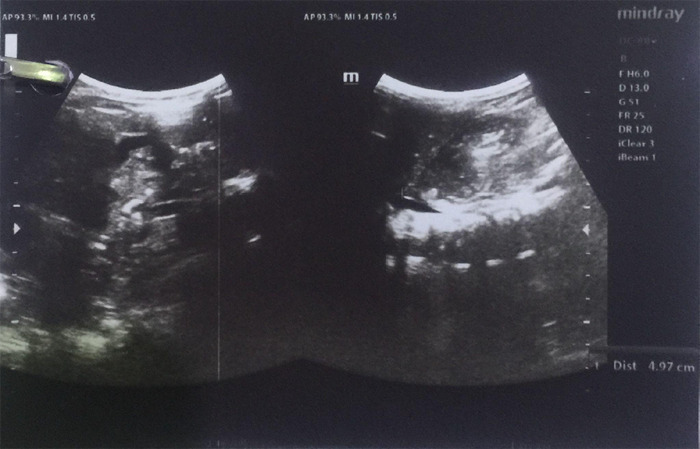
Figure 4. A) Echocardiography showing an ostium secundum ASD, B) Patent ductus arteriosus.

No gonadal abnormalities were noted. Echocardiography revealed a patent ductus arteriosus, 3.6 mm in size, and an ostium secundum-atrial septal defect (ASD), 2.6 mm with a left-to-right shunt, mild TR, and moderate pulmonary hypertension (Figure 4).

Her condition gradually improved during her stay in the ICU, leading to successful extubation. Within a short period, she no longer required oxygen support and was shifted to the ward. After completion of the antibiotic course and resolution of symptoms, she was discharged and asked for regular follow-up. During the initial follow-up visit, the patient exhibited no signs of respiratory distress. Chest X-ray showed marked improvement. A thorough hearing assessment, conducted through brainstem evoked response audiometry, revealed normal results with no abnormalities detected. We offered comprehensive counselling to the patient's family concerning her condition and the potential complications associated with her cardiac defects. The parents were advised for regular follow-up with a cardiologist until the appropriate age for surgical correction of the cardiac defects. We discussed the possibility of future spinal surgery to address the short neck and limited lateral neck bending caused by the fusion issue. We ensured that the parents were well-informed about their child's condition and the available treatment options.

## DISCUSSION

KFS is a complex condition that usually develops around early embryogenesis when a failure of segmentation of the vertebrae results in their fusion.^5^ With an average annual incidence of approximately 1 in 40000 births, it is found to affect females slightly more than males.^1,6^ Less than 50% of cases have the classic triad of features. In our report, we observed the presence of all three distinctive features in a female patient at a remarkably early age. It is a bone disorder characterized by the abnormal fusion of cervical vertebrae, along with other spinal manifestations which include scoliosis or kyphosis, Sprengel deformity, hemivertebra, basilar invagination, spina bifida, occipital-cervical synostosis, and odontoid anomalies.^7^ Along with the vertebral fusion, were able to report the presence of scoliosis in our patient. Other spinal anomalies were absent. KFS may have a wide variety of other features in addition to spine abnormalities. It includes sensorineural hearing loss, eye abnormalities, cleft palate, genitourinary problems such as abnormal kidneys or reproductive organs, cardiac abnormalities, or lung defects.^6^ Although the co-existence of multiple anomalies is quite rare, our report highlights the unique finding of two distinct associations of the renal and cardiovascular systems. Early diagnosis of these anomalies is crucial as it enables continuous monitoring of the patient's physiological response to these defects through regular follow-up appointments.

This approach is essential for tracking any changes and ensuring timely interventions if necessary.

In this case, the presence of a congenitally fused cervical segment was assessed based on plain radiographs. However, advanced imaging has not been utilized because of the cost and limited exposure to ionizing radiation. Extraspinal associations such as cross-fused renal ectopia and multiple cardiac abnormalities were noted on abdominal ultrasonography and echocardiography, respectively. Suspicion of this disease, especially at an early age, warrants a thorough investigation of all associated illnesses. Early diagnosis of these abnormalities will help provide early treatment that prevents morbidity and mortality in the future. Thorough cervical spine evaluation is essential to avoid potential spinal cord injury stemming from laryngoscopy, intubation, intraoperative positioning, and head manipulation that may increase the risk of craniovertebral dislocation and atlantoaxial subluxation.^8-10^ Furthermore, proper counselling of the parents about the course, outcome, and treatment options helps to dispel the stigma surrounding this deformity.
